# Cell-Free DNA Methylation Analysis as a Marker of Malignancy in Pleural Fluid

**DOI:** 10.21203/rs.3.rs-3390107/v1

**Published:** 2023-10-09

**Authors:** Billie Bixby, Lukas Vrba, Jyoti Lenka, Marc Oshiro, George S. Watts, Trina. Hughes, Heidi Erickson, Madhav Chopra, James L. Knepler, Kenneth S Knox, Lisa Jarnagin, Raed Alalawi, Mrinalini Kala, Richard Bernert, Joshua Routh, Denise J. Roe, Linda L. Garland, Bernard W. Futscher, Mark A. Nelson

**Affiliations:** University of Arizona; University of Arizona; University of Arizona; University of Arizona; University of Arizona; University of Arizona; University of Arizona; University of Arizona; University of Arizona; University of Arizona; University of Arizona; University of Arizona; University of Arizona; University of Arizona; University of Arizona; University of Arizona; University of Arizona

## Abstract

**Background:**

Diagnosis of malignant pleural effusion (MPE) is made by cytological examination of pleural fluid or histological examination of pleural tissue from biopsy. Unfortunately, detection of malignancy using cytology has an overall sensitivity of 50%, and is dependent upon tumor load, volume of fluid assessed, and cytopathologist experience. The diagnostic yield of pleural fluid cytology is also compromised by low abundance of tumor cells or when morphology is obscured by inflammation or reactive mesothelial cells. A reliable molecular marker that may complement fluid cytology malignant pleural effusion diagnosis is needed. The purpose of this study was to establish a molecular diagnostic approach based on pleural effusion cell-free DNA methylation analysis for the differential diagnosis of malignant pleural effusion and benign pleural effusion.

**Results:**

This was a blind, prospective case-control biomarker study. We recruited 104 patients with pleural effusion for the study. We collected pleural fluid from patients with: MPE (n = 48), PPE (n = 28), and benign PE (n = 28), and performed the Sentinel-MPE liquid biopsy assay. The methylation level of Sentinel-MPE was markedly higher in the MPE samples compared to BPE control samples (p < 0.0001) and the same tendency was observed relative to PPE (p = 0.004). We also noted that the methylation signal was significantly higher in PPE relative to BPE (p < 0.001). We also assessed the diagnostic efficiency of the Sentinel-MPE test by performing receiver operating characteristic analysis (ROC). For the ROC analysis we combined the malignant and paramalignant groups (n = 76) and compared against the benign group (n = 28). The detection sensitivity and specificity of the Sentinel-MPE test was high (AUC = 0.912). The Sentinel-MPE appears to have better performance characteristics than cytology analysis. However, combining Sentinel-MPE with cytology analysis could be an even more effective approach for the diagnosis of MPE.

**Conclusions:**

The Sentinel-MPE test can discriminate between BPE and MPE. The Sentinel-MPE liquid biopsy test can detect aberrant DNA in several different tumor types. The Sentinel-MPE test can be a complementary tool to cytology in the diagnosis of MPE.

## Introduction

A malignant pleural effusion (MPE) forms when cells from either a lung cancer or another type of cancer spread to the pleural space. These cancer cells increase the production of pleural fluid and cause decreased absorption of the fluid. People with lung cancer, breast cancer, and lymphoma (a cancer of lymphoid tissue) are most likely to get an MPE. Mesothelioma (a rare cancer of the pleura itself) is another common cause of MPE. Other causes of MPE include cancers that have spread from the stomach, kidney, ovaries, and colon^1^.

The annual incidence of pleural effusion in the United States of America is estimated to be more than 1,500,000^2^. Lung cancer is the most common cause of malignant pleural effusion accounting for approximately 1/3 of MPE cases, followed by breast cancer, ovarian cancer, and gastrointestinal cancers; the primary tumor cannot be identified in 5%-10% of MPE cases ^3,4^. Nonmalignant, benign causes of pleural effusion (BPE) include congestive heart failure, tuberculous pleuritis, pneumonia, pulmonary embolism or infarction, cirrhosis, and collagen vascular disease ^4^. A third category of PE is the paramalignant pleural effusion (PPE), whereby an effusion develops in the setting of known cancer without direct pleural involvement with tumor ^5^. Therefore, the etiology of pleural effusions has a wide differential diagnosis. A delayed diagnosis will directly affect subsequent treatment of patients and can be associated with markedly higher morbidity and mortality.

In standard care practice, cytology or pleural biopsy are typically used for diagnosing MPE ^6^. The accurate and early detection of cancer cells in the pleural effusion is of significant clinical importance in the differential diagnosis of MPE. In 50% of lung cancer cases ^7^ and 60% of all other types of cancers ^8^, the malignant characteristics of a pleural effusion can be recognized by cytology. For cytologic diagnosis of malignancy, the return of positive cancer diagnosis is highest for adenocarcinoma and lowest for mesothelioma, squamous cell carcinoma, lymphoma, and sarcoma ^4^. Repeated specimen collection with cytopathologic examination can yield an additional 27% increase in the malignancy rate ^7^. However, there are limitations to cytopathology. For example, the sensitivity of cytological analysis depends on the volume of pleural fluid sampled, the number of specimens, the type of preparation and the experience of the examiner ^9,10^. Furthermore, it is difficult to discern malignant from benign cells by morphology in the pleural fluid due to mesothelial and macrophage abnormalities. For instance, actively dividing mesothelial cells can mimic adenocarcinoma ^11^. Pleural biopsy can accurately diagnose MPE, but this procedure is invasive procedure is not routinely used because of the high risk of complications ^6^. Therefore, there is a need for new methods to diagnose malignancy in pleural fluid to prevent repeated diagnostic efforts and reduce the harm to patients.

One means to improve the diagnosis of malignant pleural effusion could be by a liquid biopsy approach. A liquid biopsy involves examining cancer-related material (i.e., cell-free DNA, protein, exosomes) from blood or other body fluids. Liquid biopsy fluids contain cell-free DNA (cfDNA) in which the circulating tumor DNA (ctDNA) fraction may be present^12–16^; the ctDNA fraction varies based on tumor type and disease progression ^17–19^. The presence of ctDNA fraction could be detected by testing cfDNA samples for tumor specific DNA methylation. DNA methylation biomarkers are more informative than DNA mutations since cancer specific DNA methylation occurs in a larger fraction of tumor samples than DNA mutations ^20^. In addition, DNA methylation can be specific to multiple cancer types that develop in different organs and tissues^16^. Since cancers have many aberrantly methylated DNA regions ^21–23^, multiple genomic loci can be investigated using DNA methylation-specific qPCR ^24^ for the presence of tumor-specific DNA methylation and thus increase sensitivity of the test.

We have developed Sentinel-MPE, a novel robust liquid biopsy assay for cancer detection. The Sentinel-MPE assay is based on the detection of tumor specific DNA methylation at ten genomic loci ^25^. Herein, we report on the diagnostic potential of the Sentinel-MPE test for the diagnosis of malignancy in PE.

## Results

### Characteristics of the Patients.

**Figure 1:**
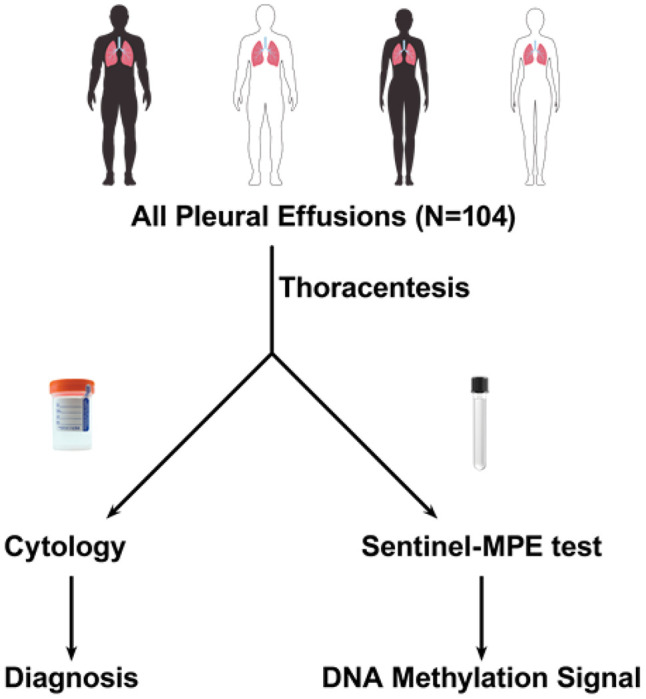
Study design for “ all comers” pleural effusion for Sentinel-MPE liquid biopsy test. This was a prospective case control study with three different cohorts of pleural effusion disease: benign pleural effusion subjects, paramalignant pleural effusion cases, and malignant pleural effusion individuals.

We prospectively collected pleural fluid from 104 patients undergoing diagnostic or therapeutic thoracentesis (Fig. 1). The study cohort included 45 males and 59 females, with a median age of 66.5 years (range 27–93). All demographic and clinical characteristics are summarized in Table 1.

### Clinicopathological Correlation Analysis.

We tested whether Sentinel-MPE DNA methylation levels were associated with the basic demographic characteristics of the patients. We did not find a significant association between Sentinel-MPE signal and patient age, gender, ethnicity or race (**eFigure, 1A-D in the Supplement**). There was a trend for African American subjects of higher methylation level (**eFigure, 1D in the Supplement**) than other racial groups. Finally, we did observe that the DNA methylation signal was significantly higher (p = 0.041) in exudative compared to transudative effusions (**eFigure, 1E in the Supplement**).

### The Diagnostic Performance of Sentinel-MPE liquid biopsy test for BPE, and all malignant PPE, and MPE cancer types.

**Figure 2:**
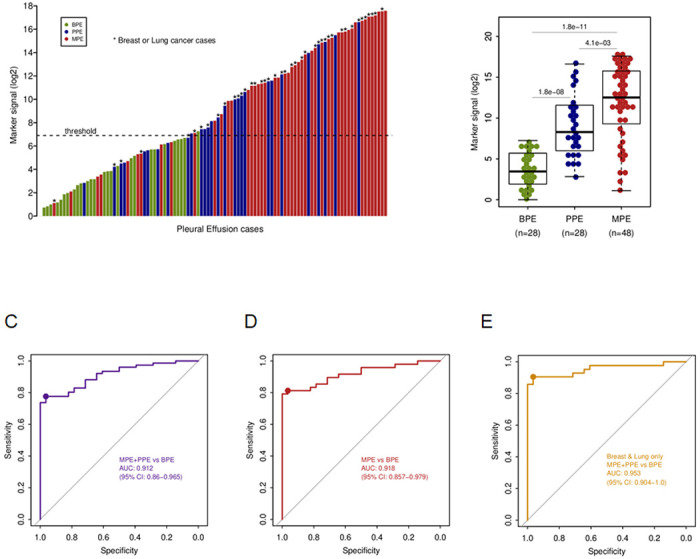
Performance of Sentinel-MPE liquid biopsy test in detecting malignancy in pleural effusion. A) Waterfall plot displaying the methylation signal from the Sentinel-MPE liquid biopsy test for all 104 participants in the study. B) Box plots displaying the mean methylation signal of benign pleural effusion (BPE), paramalignant pleural effusion (PPE) , and malignant pleural effusion cohorts (MPE). C) Receiver operating curve analysis of combined (MPE) and para-malignant effusion(PPE) groups compared to (BPE) group. The methylation signal levels were markedly higher in MPE and PPE groups relative to BPE group. D) Receiver operating curve analysis of MPE versus BPE. There was an increase in the performance of the Sentinel-MPE liquid biopsy test. E) Receiver operating curve analysis of combined MPE and PPE breast and lung cancer subjects compared to BPE subjects. Note the there was a further improvement in the performance of the Sentinel-MPE test for these two common cancer types.

We evaluated the diagnostic value of Sentinel-MPE using PE cfDNA specimens from 104 subjects. The DNA methylation level of Sentinel-MPE was significantly increased in the MPE samples compared to BPE control samples (531-fold increased median, p < 0.0001) and a similar tendency was observed relative to PPE samples (19-fold increased median, p < 0.004) (Fig. 2A–B). The DNA methylation signal was also significantly higher in PPE relative to BPE (28-fold increased median, p < 0.0001), Fig. 2B). Next, we assessed the diagnostic performance of the Sentinel-MPE test by receiver operating characteristic analysis (ROC). First, we combined the MPE and PPE groups (n = 76) and compared them against the BPE group (n = 28). As shown in Fig. 2C, the performance of the Sentinel-MPE test was high (AUC = 0.912). Second, we compared the MPE group alone against the BPE group and found an increased performance of the liquid biopsy test (AUC = 0.918) (Fig. 2D).

### The Diagnostic Performance of Sentinel-MPE liquid biopsy test for benign and malignant disease arising from Breast and Lung cancer.

Most malignant pleural effusions are secondary to metastatic involvement of the pleura from lung cancer or breast cancer ^26^. In our cohort the majority of the MPE and PPE cases were lung and breast carcinomas (42/76, 55%). We performed additional analysis with breast and lung carcinoma MPE and PPE versus BPE. The DNA methylation signal was much higher in breast and lung carcinoma cases relative to control BPE cases (414-fold increased median, p < 2.1 x 10^−13^, **eFigure, 2 in the Supplement**) and there was an additional increase in the performance of the liquid biopsy test (AUC = 0.953, Fig. 2E).

### The Diagnostic Performance of the Sentinel-MPE liquid biopsy test compared to cytology.

For all samples where data was available (n = 103, 75 malignant cases and 28 benign cases) we compared the diagnostic efficacy of the Sentinel-MPE test and traditional cytology to detect malignancy. We compared the ability of cytology alone, the Sentinel-MPE test, and both tests combined to detect malignancy in pleural effusion. Cytology analysis had a sensitivity of 49% (Table 2), whereas the Sentinel-MPE liquid biopsy test had a sensitivity of 76% (Table 2). There is further improvement in identifying malignant disease by combining both tests (sensitivity 84%, Table 2). This data indicates that the use of pleural effusion cfDNA methylation analysis using the Sentinel-MPE test could facilitate the early diagnosis of malignancy on the initial specimen collected.

## Discussion

Malignant pleural effusion (MPE) is a frequent clinical problem in patients with neoplasia and represents advanced malignant disease with a poor prognosis with limited therapeutic options if not identified early. Patients with BPE can be managed with interventions that frequently provide resolution of the benign effusion or long-term control, with attention to the underlying benign etiology. Thus, it is essential to accurately distinguish between BPE and MPE for therapeutic decisions and to improve the prognosis of patients with MPE. Although there have been advancements in new imaging modalities, confirmation of malignant cells in the PE or pleural biopsy is necessary to establish a definitive diagnosis of MPE. Compared to pleural biopsy, cytological examination of pleural fluid represents a much less invasive procedure. However, cytology has limited diagnostic sensitivity (between 50–60%) due to substantial overlapping morphologic features among malignant, mesothelial, and reactive cells ^27^. Pleural fluid is a source for liquid biopsy applying novel analyses to aid in the diagnosis of MPE. Here we report the development of molecular diagnostic assay to address these issues.

In agreement with previous studies^28,29^, we demonstrate that pleural fluid can be a sample source of liquid biopsy for the detection of malignancy. Lung cancer and metastatic breast cancer are two major causes of pleural effusion ^26^. In the present study, we also showed that the Sentinel-MPE test could detect aberrant DNA methylation in both non-small cell lung cancer (NSCLC) and breast cancer cases with high sensitivity and specificity. An independent case control study of breast and lung cancer cases is currently underway.

Almost all cancers can potentially have pleural involvement causing MPE and many of them originate from cancer metastases ^2,5^ making a test that can detect a variety of cancer type is deal. In the present study, we demonstrate that the Sentinel-MPE liquid biopsy test could detect malignancy in pleural effusions from several different cancer type such as: AML, colon cancer, lymphoma, and ovarian carcinomas.

Blood and sputum represent the two primary biofluid sources for liquid biopsy in the oncology literature. Pleural fluid, as a novel source, has significant advantages due to direct access to malignant cells and their microenvironment. Continued access to pleural fluid also allows characterization of targetable mutations for some patients ^30^. Our study indicates that a molecular diagnostic test, based on DNA methylation analysis, could be of value in identification of MPE which could impact patient care.

Potential limitations to our study include the modest sample and studies with more patients are needed to validate the results in the future. Another limitation of the Sentinel-MPE test is that the assay did not detect sarcomas and G.I. neuroendocrine tumors Additional bioinformatics studies with genome wide methylation datasets are needed to identify DNA methylation markers for ovarian, sarcomas, and neuroendocrine cancers to include in the Sentinel-MPE test. Our group is conducting these in silico studies. Finally, whether the methylation changes of the Sentinel-MPE test correlate with the prognosis of MPE patients requires further study.

The differentiation between MPE and PPE is important to ensure appropriate patient management ^31^ as finding MPE in patients with known cancer may upstage the cancer, thus leading to different therapeutic strategies such as curative versus palliative treatment In the present study, we noted a significant difference in the methylation signal between MPE and PPE. However, further studies are warranted to determine if such methylation signal differences can be exploited to discern MPE from PPE.

## Conclusions

The results from this study indicate that the Sentinel-MPE test displayed desirable performance characteristics in differentiating MPE from BPE. In addition, the Sentinel-MPE can detect aberrant DNA methylation in several different tumor types such as NSCLC, breast cancer, lymphoma, and acute myeloid leukemia, among others. Importantly, the Sentinel-MPE can be a complementary tool for the cytopathologist to improve on the sensitivity and specificity of cytology alone in the diagnosis of MPE.

## Methods

### Study Design and Participants.

We performed a prospective case-control study (Fig. 1). We accrued pleural effusion samples from patients admitted to the hospital or seen in the pulmonology clinics at a single academic medical center The University of Arizona Human Subjects Protection Program approved the study and each participant provided written informed consent. Diagnostic cohort classifications were assigned by the study Principal Investigator as follows: Patients with no documented cancer were classified as having benign pleural effusion (BPE). Patients determined to have pleural involvement of cancer by cytology, biopsy, imaging, or clinical assessment were classified as having malignant pleural effusion (MPE). Patients with cancer that did not have clinical/radiographic evidence of pleural involvement by cancer were classified as having paramalignant pleural effusion (PPE). Patient medical records were reviewed for pleural effusion cytology. Pleural effusion fluid samples were collected using Streck Cell-Free DNA BCT tubes (La Vista, NE) for DNA methylation analysis. The samples were assigned a study number and submitted to the laboratory in a blinded fashion. Cytology results were confirmed by two pathologists who were blinded to the Sentinel-MPE test results.

### DNA extraction and bisulfite treatment.

Cell-free DNA was extracted from 2.0 ml samples of pleural effusion fluid as previously described by our group^25^. The quantity of cfDNA was assessed by Qubit and Nanodrop instruments. Up to 500 ng of cfDNA was used for bisulfite treatment performed using EZ DNA Methylation-Gold Kit (Zymo Research, Irvine, CA, USA) as previously described^25^.

### DNA methylation analysis.

The two-step methylation specific quantitative PCR was performed on QuantStudio 5 Real-Time PCR instrument using parameters we described before^25^.

### Statistical Analysis.

Statistical analyses were performed in R programming environment ver 4.3.1 (R Foundation for Statistical Computing, Vienna, Austria). Since the DNA methylation signal from the biomarkers spans several orders of magnitude, nonparametric tests were used to test differences between the groups (Wilcoxon rank sum test) or correlation between variables (Spearman’s rank correlation coefficient). A two-sided *p* value < 0.05 was considered statistically significant. Receiver operating characteristic (ROC) analysis was performed using R library pROC to determine the areas under the curve (AUC) including 95% DeLong confidence intervals (CIs) and analytical threshold for the best accuracy (DNA methylation signal for the point at the ROC curve closest to the upper left corner (1.0,1.0)).The analytical threshold was used to classify samples as benign (DNA methylation signal bellow the threshold) or malignant (DNA methylation signal above the threshold) and to determine accuracy, sensitivity, specificity, positive predictive value (PPV) and negative predictive value (NPV) of the Sentinel-MPE assay (R library caret). For the combined assay of Sentinel-MPE and cytology, samples positive in either assay were considered positive, and samples negative in both assay classified as negative. The 95% Wilson score-based confidence intervals for the assay accuracy, sensitivity and specificity were determined.

## Figures and Tables

**Table 1:** Demographic and Characteristics of Patients

	Benign	Malignant	PPE
**Age (years)**			
Mean +/− SD	62.8 +/− 17.7	63.8 +/− 14.4	68 +/− 12.7
Range	29-93	27-83	27-92

**Gender**			
Male (45, *43%)*	14 (50%)	20 (42%)	11 (39%)
Female (59, *57%)*	14 (50%)	28 (58%)	17 (61%)

**Tobacco use**			
History (60, *58%)*	19 (68%)	24 (50%)	17 (61%)
Never (44, *42%)*	9 (32%)	24 (50%)	11 (39%)

**Malignancy**			
Lung		20	13
Breast		8	3
Hematologic		5	2
Ovarian		4	-
Uterine		2	2
Head/neck SCC		-	2
Hepatobiliary		1	1
GI NET		1	1
Sarcoma		2	-
Esophageal		-	1
Gastric		1	-
Germ cell		1	-
Thyroid		1	-
SFT high risk		-	1
EHE		-	1
Schwannoma Mesothelioma*		1	-
Unknown**		1	-
		1	-

**BPE**			
Infection	9		
Heart Failure Hemothorax	6		
Trapped lung	4		
Renal failure	4		
Cirrhosis	3		
VP shunt	1		
	1		

MPE: malignant pleural effusion; PPE/IPE: paramalignant effusion/indeterminate pleural effusion in a subject with known cancer; SCC: squamous cell carcinoma; GI NET: gastrointestinal neuroendocrine tumor; SFT: solitary fibrous tumor; EHE: epithelioid hemangioendothelioma, BPE: benign pleural effusion; VP: ventricular peritoneal.

* peritoneal

** adenocarcinoma of unknown primary

**Table 2:** Diagnostic performance of Sentinel-MPE test for BPE and MPE.

Test	Accuracy (95% CI)	Sensitivity (95% CI)	Specificity (95% CI)	Positive Predictive Value (PPV) (95% CI)	Negative Predictive Value (NPV) (95% CI)
**Cytology**	0.631 (0.534–0.718)	0.493 (0.383–0.604)	1.000 (0.854-1.000)	1.000 (0.885-1.000)	0.424 (0.313-0.545)
**Sentinel-MPE**	0.816 (0.728–0.879)	0.760 (0.651–0.843)	0.964 (0.805-1.000)	0.983 (0.898-1.000)	0.600 (0.454–0.729)
**Cytology+ Sentinel-MPE**	0.874 (0.794–0.926)	0.840 (0.739–0.907)	0.964 (0.805-1.000)	0.984 (0.907-1.000)	0.692 (0.534–0.814)

## Data Availability

Not applicable

## Abbreviations

AUC: Area under the curve
ROC: Receiver operating characteristic
cfDNA: Cell-free DNA
BPE: Benign pleural effusion
MPE: Malignant pleural effusion
PPE: Parmalignant pleural effusion
PPV: Positive predictive value
NPV: Negative predictive value
CI: confidence intervals

## References

[1] Semaan R, Feller-Kopman D, Slatore C, Sockrider M. Malignant Pleural Effusions. Am J Respir Crit Care Med 2016;194:P11–2. 27628085 10.1164/rccm.1946P11

[2] Bashour SI, Mankidy BJ, Lazarus DR. Update on the diagnosis and management of malignant pleural effusions. Respir Med 2022;196:106802. 35287006 10.1016/j.rmed.2022.106802

[3] Jiang B, Li XL, Yin Y, Ultrasound elastography: a novel tool for the differential diagnosis of pleural effusion. Eur Respir J 2019;54. 10.1183/13993003.02018-2018 31151959

[4] Jany B, Welte T. Pleural Effusion in Adults-Etiology, Diagnosis, and Treatment. Dtsch Arztebl Int 2019;116:377–86. 31315808 10.3238/arztebl.2019.0377 PMC6647819

[5] Sahn SA. Pleural diseases related to metastatic malignancies. Eur Respir J 1997;10:1907–13. 9272937 10.1183/09031936.97.10081907

[6] Skok K, Hladnik G, Grm A, Crnjac A. Malignant Pleural Effusion and Its Current Management: A Review. Medicina (Kaunas) 2019;55. 10.3390/medicina55080490 PMC6723530 31443309

[7] Asciak R, Rahman NM. Malignant Pleural Effusion: From Diagnostics to Therapeutics. Clin Chest Med 2018;39:181–93. 29433714 10.1016/j.ccm.2017.11.004

[8] Hooper C, Lee YC, Maskell N, Group BTSPG. Investigation of a unilateral pleural effusion in adults: British Thoracic Society Pleural Disease Guideline 2010. Thorax 2010;65 Suppl 2:ii4–17. 20696692 10.1136/thx.2010.136978

[9] Porcel JM, Light RW. Pleural effusions. Dis Mon 2013;59:29–57. 23374395 10.1016/j.disamonth.2012.11.002

[10] Woenckhaus M, Grepmeier U, Werner B, Microsatellite analysis of pleural supernatants could increase sensitivity of pleural fluid cytology. J Mol Diagn 2005;7:517–24. 16237222 10.1016/S1525-1578(10)60583-1 PMC1888495

[11] Dietrich D, Jung M, Puetzer S, Diagnostic and prognostic value of SHOX2 and SEPT9 DNA methylation and cytology in benign, paramalignant and malignant pleural effusions. PLoS One 2013;8:e84225. 24386354 10.1371/journal.pone.0084225 PMC3874014

[12] Schwarzenbach H, Hoon DS, Pantel K. Cell-free nucleic acids as biomarkers in cancer patients. Nat Rev Cancer 2011;11:426–37. 21562580 10.1038/nrc3066

[13] Wan JCM, Massie C, Garcia-Corbacho J, Liquid biopsies come of age: towards implementation of circulating tumour DNA. Nat Rev Cancer 2017;17:223–38. 28233803 10.1038/nrc.2017.7

[14] Leon SA, Shapiro B, Sklaroff DM, Yaros MJ. Free DNA in the serum of cancer patients and the effect of therapy. Cancer Res 1977;37:646–50. 837366

[15] Snyder MW, Kircher M, Hill AJ, Daza RM, Shendure J. Cell-free DNA Comprises an In Vivo Nucleosome Footprint that Informs Its Tissues-Of-Origin. Cell 2016;164:57–68. 26771485 10.1016/j.cell.2015.11.050 PMC4715266

[16] Moss J, Magenheim J, Neiman D, Comprehensive human cell-type methylation atlas reveals origins of circulating cell-free DNA in health and disease. Nat Commun 2018;9:5068. 30498206 10.1038/s41467-018-07466-6 PMC6265251

[17] Bettegowda C, Sausen M, Leary RJ, Detection of circulating tumor DNA in early-and late-stage human malignancies. Sci Transl Med 2014;6:224ra24. 10.1126/scitranslmed.3007094 PMC4017867 24553385

[18] Diehl F, Li M, Dressman D, Detection and quantification of mutations in the plasma of patients with colorectal tumors. Proc Natl Acad Sci U S A 2005;102:16368–73. 16258065 10.1073/pnas.0507904102 PMC1283450

[19] Jahr S, Hentze H, Englisch S, DNA fragments in the blood plasma of cancer patients: quantitations and evidence for their origin from apoptotic and necrotic cells. Cancer Res 2001;61:1659–65. 11245480

[20] Zou H, Allawi H, Cao X, Quantification of methylated markers with a multiplex methylation-specific technology. Clin Chem 2012;58:375–83. 22194633 10.1373/clinchem.2011.171264

[21] Novak P, Jensen T, Oshiro MM, Watts GS, Kim CJ, Futscher BW. Agglomerative epigenetic aberrations are a common event in human breast cancer. Cancer Res 2008;68:8616–25. 18922938 10.1158/0008-5472.CAN-08-1419 PMC2680223

[22] Rauch TA, Zhong X, Wu X, High-resolution mapping of DNA hypermethylation and hypomethylation in lung cancer. Proc Natl Acad Sci U S A 2008;105:252–7. 18162535 10.1073/pnas.0710735105 PMC2224196

[23] Shames DS, Girard L, Gao B, A genome-wide screen for promoter methylation in lung cancer identifies novel methylation markers for multiple malignancies. PLoS Med 2006;3:e486. 17194187 10.1371/journal.pmed.0030486 PMC1716188

[24] Eads CA, Danenberg KD, Kawakami K, MethyLight: a high-throughput assay to measure DNA methylation. Nucleic Acids Res 2000;28:E32. 10734209 10.1093/nar/28.8.e32 PMC102836

[25] Vrba L, Oshiro MM, Kim SS, DNA methylation biomarkers discovered in silico detect cancer in liquid biopsies from non-small cell lung cancer patients. Epigenetics 2020;15:419–30. 31775567 10.1080/15592294.2019.1695333 PMC7153541

[26] Shojaee S, Sharma A, Gottel N, Sanchez T, Gilbert JA, Rahman NM. Microbiome profile associated with malignant pleural effusion. PLoS One 2020;15:e0232181. 32384089 10.1371/journal.pone.0232181 PMC7209204

[27] Kaul V, McCracken DJ, Rahman NM, Epelbaum O. Contemporary Approach to the Diagnosis of Malignant Pleural Effusion. Ann Am Thorac Soc 2019;16:1099–106. 31216176 10.1513/AnnalsATS.201902-189CME

[28] Liang C, Liu N, Zhang Q, A detection panel of novel methylated DNA markers for malignant pleural effusion. Front Oncol 2022;12:967079. 36176402 10.3389/fonc.2022.967079 PMC9513209

[29] Zhang C, Yu W, Wang L, DNA Methylation Analysis of the SHOX2 and RASSF1A Panel in Bronchoalveolar Lavage Fluid for Lung Cancer Diagnosis. J Cancer 2017;8:3585–91. 29151944 10.7150/jca.21368 PMC5687174

[30] Mahmood K, Jampani P, Clarke JM, High Yield of Pleural Cell-Free DNA for Diagnosis of Oncogenic Mutations in Lung Adenocarcinoma. Chest 2023;164:252–61. 36693563 10.1016/j.chest.2023.01.019 PMC10331627

[31] Arora RD, Boster J. Malignant Pleural Effusion. StatPearls. Treasure Island (FL) ineligible companies. Disclosure: Joshua Boster declares no relevant financial relationships with ineligible companies.2023.

